# Evaluation of the Performance of Information Theory-Based Methods and Cross-Correlation to Estimate the Functional Connectivity in Cortical Networks

**DOI:** 10.1371/journal.pone.0006482

**Published:** 2009-08-04

**Authors:** Matteo Garofalo, Thierry Nieus, Paolo Massobrio, Sergio Martinoia

**Affiliations:** 1 Neuroscience and Brain Technology Department, Italian Institute of Technology, Genova, Italy; 2 Neuroengineering and Bio-nano Technology Group (NBT), Department of Biophysical and Electronic Engineering (DIBE), University of Genova, Genova, Italy; Indiana University, United States of America

## Abstract

Functional connectivity of *in vitro* neuronal networks was estimated by applying different statistical algorithms on data collected by Micro-Electrode Arrays (MEAs). First we tested these “connectivity methods” on neuronal network models at an increasing level of complexity and evaluated the performance in terms of ROC (Receiver Operating Characteristic) and PPC (Positive Precision Curve), a new defined complementary method specifically developed for functional links identification. Then, the algorithms better estimated the actual connectivity of the network models, were used to extract functional connectivity from cultured cortical networks coupled to MEAs. Among the proposed approaches, Transfer Entropy and Joint-Entropy showed the best results suggesting those methods as good candidates to extract functional links in actual neuronal networks from multi-site recordings.

## Introduction

**Figure 1:**
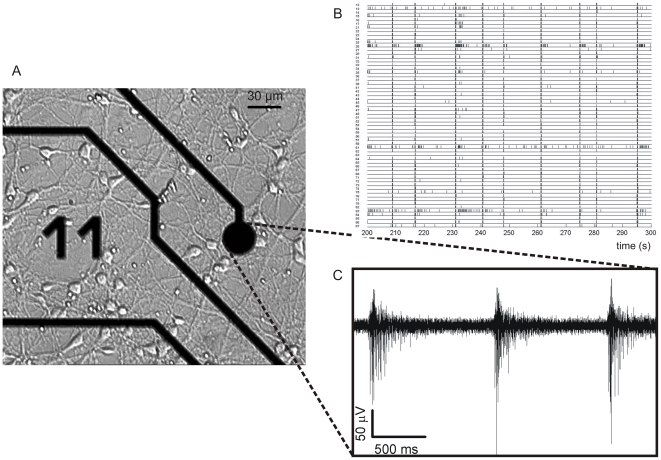
MEA and recorded signals overview. (A) Dissociated cortical neurons coupled to a MEA. (B) Raster plot of the electrophysiological activity: each row corresponds to a recording site, and each small vertical line corresponds to a detected spike. (C) Electrophysiological activity recorded from one microelectrode.

Large random networks of cortical neurons developing *in vitro* and chronically coupled to Micro-Electrode Arrays (MEAs) (Figure 1A) represent a well established experimental model for studying the neuronal dynamics at the network level [1]–[5], and for understanding the basic principles of information coding [6], separation property [7], learning [8], and memory [9]. These preparations, unlike other experimental models such as acute and cultured cortical slices, are relatively free of predefined constraints and allow neurons to establish several synaptic links, creating highly-connected networks which exhibit complex dynamics characterized by highly-synchronized bursts and random spiking (Figures 1B and 1C).

The introduction of MEAs allows simultaneous recordings from tens of microelectrodes, giving the opportunity to access several “nodes” of the network, to study how neurons are connected each other, and which topological architectures underlie a specific dynamic behavior [10], [11]. Within this topic, recent technological efforts (increase of the number of electrodes and of the spatial resolution [12]), allow to obtain a more precise mapping of the neuronal network up to a possible identification of its anatomical connections (i.e., the set of physical or structural-synaptic connections linking neuronal units at a given time [13]). Thus, to better understand the neuronal dynamics of a wide variety of complex networks, it becomes fundamental to investigate how neurons are functionally connected. Within this general framework, several approaches can be followed, depending on the scale at which the nervous system is observed. Functional imaging or optical methods, as fluorescent techniques, could be a possible strategy to achieve such goal for *in vitro* preparations. However, there are some drawbacks related to the limited access to single units and large populations at the same time, and to a poor temporal resolution [14].

A different approach relies on the identification of causal relationships between pairs of neurons by means of electrophysiological measurements: this complementary method plays a relevant role in the study of synaptic interactions at microcircuit and at population level.

Nowadays, a promising technique to infer connectivity maps of a cell culture seems to rely on an investigation of the statistical properties of the spontaneous activity. This technique, also called functional and effective connectivity method, relies on the pair-wise spiking activities of the neurons.

Functional connectivity [13], [15] captures patterns of deviations from statistical independence between distributed neuronal units, measuring their correlation/covariance, spectral coherence or phase-locking. Functional connectivity is often evaluated among all the elements of a system, regardless whether these elements are connected by direct structural links; moreover, it is highly time-dependent (hundreds of milliseconds) and model-free, and it measures statistical interdependence (e.g. mutual information) without explicit reference to causal effects.

On the other hand, effective connectivity [16] describes the set of causal effects of one neuronal system over another one, either directly or indirectly. Thus, unlike functional connectivity, effective connectivity is not model-free, but it requires the specification of a causal model which includes structural parameters. Experimentally, effective connectivity can be inferred by perturbations or by the observation of the temporal ordering of neuronal events. Obviously, anatomical links play a critical role in determining which functional or effective connections can and cannot occur.

In this work, we used correlation and information theory-based methods to estimate the functional connectivity of *in vitro* neuronal networks. The rationale consists in applying such methods to each possible pair of electrodes which shows spontaneous electrophysiological activity. The connection strength (described by means of the synaptic weight) between two neurons is supposed to be proportional to the value yielded by the method.

In particular, we used, Mutual Information (MI) [17], Joint-Entropy (JE) and Transfer Entropy (TE) [18] methods, compared to the standard and well known Cross-Correlation (CC) [19]. CC measures the frequency at which one cell fires as a function of time relative to the firing of a spike in another cell. MI represents a measure of the statistical dependence between two spike trains recorded by two microelectrodes. JE, here introduced for the first time in the field of Neuroscience, is a linear method as CC, but it is built by considering the cross inter-spike-intervals (cISI) computed across pairs of neurons. Measures based on cISI histograms are well-known in the literature, also in the functional connectivity investigation on simultaneous recorded spike trains [20]. Then, cISI analysis was used on small networks (characterized by few neurons and few connections) to uncover inhibitory connections (which are expected to delay or even to take out spike generation), as well to detect the temporal integration of successive stimulations [21]. TE is an information theoretic measure able to estimate causal relationships from time series taking into account their past activity. In particular, TE estimates the part of a neuron activity which does not depend on its own past, but which depends on another neuron's past activity. Moreover, TE takes into account linear and nonlinear interactions, and thus it can represent a general way to define the causality strength between two spikes trains.

However, it is known [22] that inferring the strength of a synaptic contact by just considering the pair-wise activities may be difficult because, in the considered neuronal systems, cells are highly connected, i.e., cells contact each others either directly (by a single synaptic connection) or indirectly (by a di-tri or even longer synaptic connection pathways). More recently, higher-point mutual information techniques have been devised to dissect the contribution of indirect pathways. In [23], the pair-wise activity of two neurons is evaluated compared to a third one by means of a redundancy measure. Although higher-point mutual information techniques seem to be promising, no effort has been made to test and validate the obtained connectivity maps on highly connected neuronal assemblies. Recently [24], a Granger causality based approach has been shown to be useful to recover the synaptic strengths in a simplified neuronal network, but to our knowledge, no attempts have been made to validate connectivity methods on wider and more realistic neuronal networks.

The purpose of this work is to address this issue by applying standard and *ad hoc* procedures to evaluate the performance of the connectivity methods on realistic models of neuronal networks. To achieve this goal, we built synthetic spike trains originated by the simulations of neuronal network models made up of spatially distributed and synaptically connected neurons (described by the Izhikevich equations [25]), and we represented the topology of these neuronal network models by a Synaptic Weight Matrix (SWM).

Performances were estimated by ROC (Receiver Operating Characteristic) [26] and by a new defined complementary method named PPC (Positive Precision Curve). ROC, widely used in the signal theory, is useful for assessing the accuracy of a prediction. However, the discriminating capabilities among different methods are practically reduced to a single scalar value defined as the Area Under the Curve (AUC). In addition to the coarse AUC parameter, PPC allows to evaluate not only the absolute number of the existing/non-existing connections identified by the connectivity methods but also to recognize the synaptic weights following the right ordering. Therefore, PPC adds further information regarding how the connectivity methods identify the connections.

## Materials and Methods

### Cell culture, experimental set-up and data analysis

Dissociated cortical neurons were extracted from rat embryos and plated on 60-channel MEAs precoated with adhesion promoting molecules (poly-D-lysine and laminin), at the final density of 5−8×10^4^ cells/device, which means about 1200–1400 cells/mm^2^. They were maintained in culture dishes, each containing 1 ml of nutrient medium (i.e. serumfree Neurobasal medium supplemented with B27 and Glutamax-I, [27]) and placed in a humidified incubator having an atmosphere of 5% CO_2_ and 95% O_2_ at 37°C. Under these environmental conditions, cortical neurons showed excellent growth and robust synaptic connections that allowed us to record spontaneous electrical activity from 7 days *in vitro* (DIV) up to 5–6 weeks *in vitro* (WIV). Further details about cell cultures can be found in [4].

The network electrophysiological activity was recorded after the third-fourth WIV to allow the maturation of synaptic connections among the cells of the network.

The experimental set-up was based on the MEA60 System (Multi Channel Systems, MCS, Reutlingen, Germany).

The electrophysiological activity was recorded without any chemical or electrical stimulation (i.e., it was referred only to the spontaneous activity). The recorded signals ranged from random spike activity to more complicated and synchronized burst signals (Figures 1B and 1C).

Extracellular recorded signals were embedded in biological and thermal noise. These raw signals were recorded and sampled at 10 kHz, and data were then processed off-line by using custom software. Spiking and bursting activities were identified by using a spike detection algorithm [28]. The previously validated algorithm is based on a Differential Threshold (DT) for each channel, and it is used to discriminate population spike events. Briefly, after setting the threshold to 8 times the standard deviation of the biological noise [29], [30], the algorithm considers a portion of the signal and looks for the Relative Maximum/Minimum whose peak-to-peak amplitude is above the defined threshold. Then, a candidate spike undergoes additional checks, such as the peak lifetime period (set as 2 ms) and the refractory period (set as 1 ms), in order to ensure the correct identification of a spike and its precise timing.

From the spike trains, we evaluated the common metrics used to characterize both simulated and experimental data. In particular, we computed the Inter-Spike-Interval (ISI), the Inter-Burst-Interval (IBI), the Mean Firing Rate (MFR) and the Mean Bursting Rate (MBR) [6].

### Network Model

We implemented a neuronal network model mimicking the electrophysiological activity of cultured cortical neurons under spontaneous conditions.

Following the approach proposed by Izhikevich [25], we developed a neuronal network model made up of 60 spatially distributed and synaptically connected neurons. To test the above mentioned algorithms, we implemented different network configurations at an increasing level of complexity and similarity with the biological networks. The results presented in this work are related to two main configurations.

Firstly, we implemented a simple neuronal network with only excitatory connections and synaptic weights extracted from a normal distribution. We named this configuration *E*. Secondly, we developed a more physiological network model including also inhibitory connections; we named this configuration *EI*. In this configuration, we considered two different types of neurons to model excitatory and inhibitory populations: the former type belongs to the family of regular spiking neurons, and the latter to the family of fast spiking neurons [25], [31]. Regular spiking neurons fire with a few spikes characterized by short ISI at the onset of an input. Differently, fast spiking neurons exhibit periodic trains of action potentials at higher frequencies without adaptation. To preserve the main characteristics of the structure of the *in vitro* cortical neurons, we set the ratio between excitatory and inhibitory neurons to 4∶1 [8], [9], [32]. These two families of neurons were connected in a random way with the constraint that each neuron can be connected (outgoing connections) to a maximum number of other neurons. The most relevant parameters relative to *E* and *EI* networks are summarized in Table 1.

**Table 1:** Network model parameters considered in the simulations.

Model Name	Synaptic weights (exc; inh)	Number of neurons (exc; inh)	Max # of outgoing connections
E	4÷13; 0÷0	60; 0	23
EI	4÷16; −4÷−16	48; 12	30

Synaptic weights are chosen randomly, with a uniform distribution, in the reported interval (e.g. 4÷13 for excitatory connections of Model E). Positive (negative) weights correspond to an excitatory (inhibitory) synaptic connection.

Spontaneous activity was obtained by introducing a randomly distributed stimulation reproducing the effect of fluctuation in the membrane potential [33] due to the distributed background activity.

All the simulations, performed in Matlab environment (The Mathworks, Natick MA, USA), lasted 300 s at 0.1 ms integration time-step. The simulation output was then peak-detected by means of a simple hard-threshold algorithm.

To assess the stability of the considered connectivity methods, we simulated 5 *EI* and *E* configurations, obtained by changing the network configuration (i.e., the seed generating synaptic pathways and weights). By averaging the MFR and MBR over the 5 realizations, we found a MFR = 11.1±0.7 spikes/s (mean±standard deviation) and a MBR = 8.7±1.0 bursts/min for the *E* configuration model, and a MFR = 7.9±0.5 spikes/s and a MBR = 15.8±1.1 bursts/min for the *EI* configuration model. In order to verify that the dynamics of the *EI* configuration fit the experimental data, we evaluated the network histograms ISI and IBI (data not shown). ISI histograms showed a Poisson-like distribution, and IBI distributions were characterized by marked peaks in the first bins.

This dataset was used to test the performance of the connectivity methods detailed in the next sections.

### Connectivity methods

**Figure 2:**
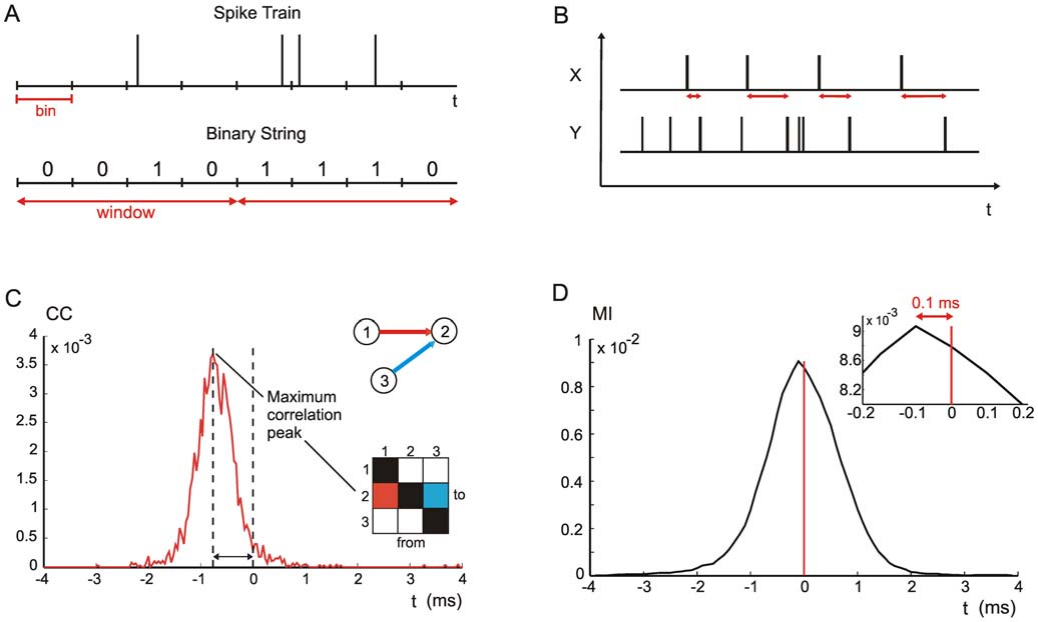
Schematic overview of the considered connectivity methods. (A) Binary string is created starting from the spike train. A window is selected to evaluate the TE and to define the MI symbols (window = 0.3 ms). (B) Cross-inter-spike-intervals (cISI) between neurons *X* and *Y* are highlighted by the red arrows. (C) Cross-correlation function between neuron 1 and 2. The directionality of the connection is evaluated considering the peak latency from zero. (D) Mutual information (spike count approach) function related to a pair of nodes of the network model. The inset shows that the MI peak value falls close to the zero time shift (value −0.1 ms).

Connectivity maps of neuronal networks can be inferred on the basis of the spiking activity of single neurons. This approach, also known as functional connectivity, analyzes the series of spike train timestamps (Figure 2). Functional connectivity was estimated by using the above-introduced four methods: CC, MI, JE, and TE. For each pair of neurons, the connectivity method provides an estimation of the connection strength (one for each direction). Thus, each method is associated to a matrix, the Connectivity Matrix (CM), whose elements (*X*, *Y*) correspond to the estimated connection strength between neuron *X* and *Y*.

**Figure 3:**
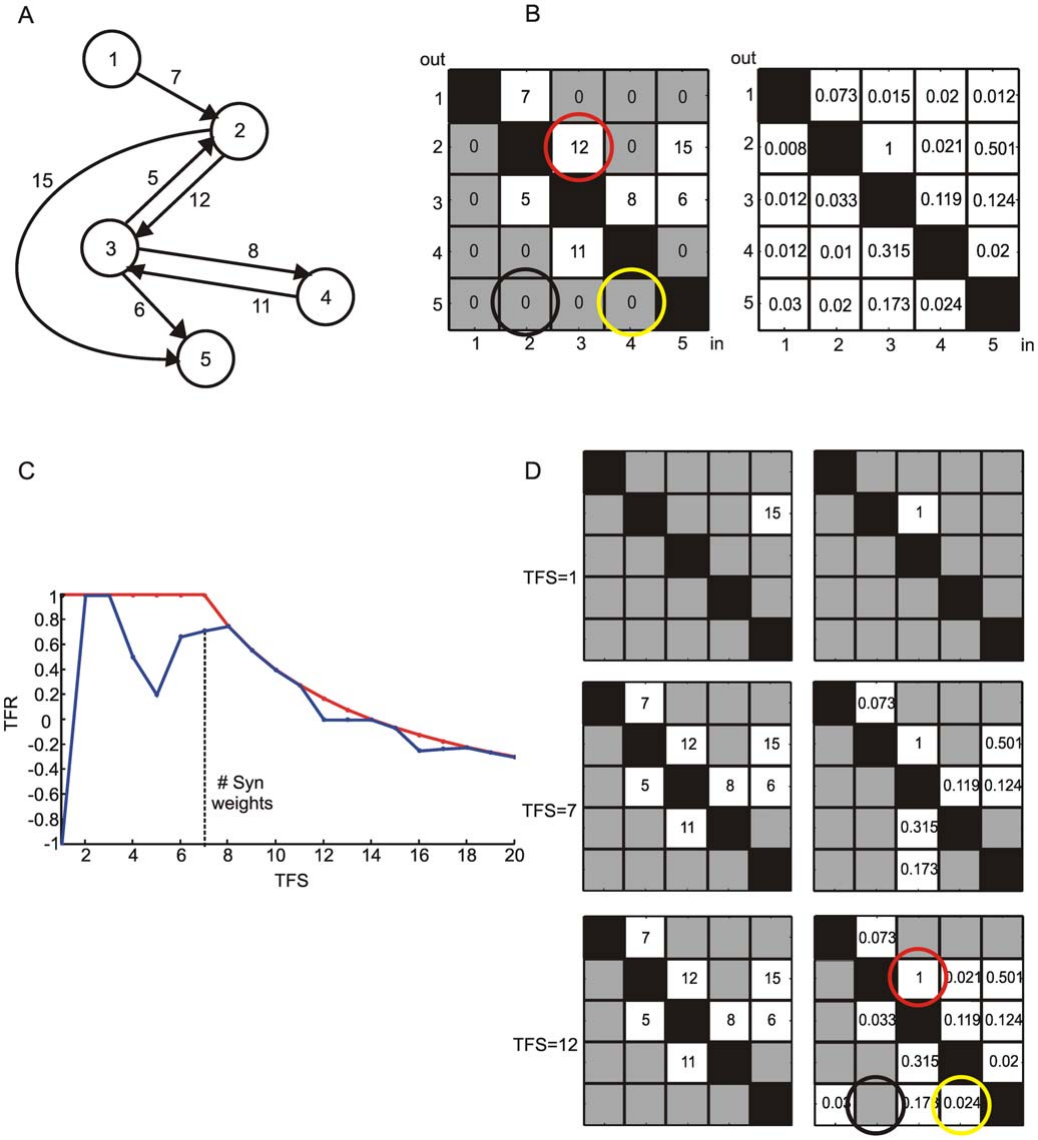
PPC working principle. A small network consisting of five neurons is considered. (A) Network graph. The numbers indicated on the arrows are the synaptic weights. (B) SWM (left) and CM (right). The red, black and yellow circles on the TCM correspond to a true positive (TP), true negative (TN) and false positive (FP), respectively. (C) Positive Precision Curve. The red curve corresponds to the best performance a given method could achieve. The dashed black line corresponds to the number of synaptic weights present in the model (panel A). (D) By comparing the thresholded SWM (left) to the TCM (right) the blue curve (drawn as an example) of the positive precision plot (panel C) is obtained. The white background elements on the TCMs (right) correspond to the TFS elements being analyzed.

High and low values in the CM are expected to correspond to strong and weak connections. By using such approach, inhibitory connections could not be detected because they would be mixed with small connection values. However, non-zero CM values were also obtained when no apparent causal effects were evident, or no direct connections were present among the considered neurons. In principle, by thresholding the CM, it would be possible to filter out the noisy and non-causal values (because they are expected to be small). Anyhow, for each threshold value, a connectivity map is obtained. These maps, deduced by considering only the strongest CM values, display the links which should correspond to the strongest synaptic pathways. An exemplificative case is shown in Figure 3. The CM is displayed in Figure 3B (right) and some corresponding Thresholded Connectivity Matrix (TCM) are reported in Figure 3D (right).

#### Cross-Correlation function

Cross-Correlation (CC) function [19] was built by considering the spike trains (Figure 2A) of two neurons. It measures the frequency at which one cell fires (‘target’) as a function of time, relative to the firing of a spike in another cell (‘reference’). Mathematically [34], CC reduces to a simple probability *C_xy_*(*τ*) of observing a spike in a train *Y* at time (*t+τ*), because of a spike in another train *X* at time *t* [6]; τ is called time shift or time lag.

CC function was evaluated considering all the pairs of spike trains. Connection strength among neurons was evaluated on the basis of the peak value of the CC function. Therefore, CM is defined by considering the peak values of each CC function: the highest CC values should correspond to the strongest connections. Additionally, directionality was deduced from the sign of the corresponding peak latency (Figure 2C).

To compute CC function, the time lag was set to 0.1 ms and the time frame was set to 150 ms.

#### Mutual Information

Mutual Information (MI) is a measure of the statistical dependence between two processes. To compute MI between two neurons, spike trains were represented as binary strings. Time is discretized, such that each time bin (0.1 ms, Figure 2A) represents either the presence or the absence of a spike. Successive time bins are aggregated to the extent of a fixed time window (i.e., a binary string) in order to translate the entire spike train into a sequence of binary symbols. Depending on which mode of coding mechanism we are interested in, the binary strings are regarded as temporal patterns (time code) or translated by counting the spike occurrences (spike count code, e.g. in Figure 2A ‘1110’ correspond to 3 spikes). In principle, a time code approach should give the best results. However, preliminary tests indicated the best performances in terms of recovering connectivity maps were obtained by considering small temporal windows. We varied both the bin size and the time window of MI identifying an optimal time window equal to a bin size of 0.3 ms (cf., “Bin choice for the parametric methods” in the Results section). Considering such time scale, the same tests also indicated that time and spike code approaches behave similarly (data not shown).

MI is computed by evaluating joint and single probabilities of the two neurons (*X*,*Y*):
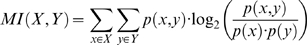
(1)where *x*, *y* represent a single event (e.g., *x* = 2, *y* = 3 spikes). Each joint probabilities, *p*(*x,y*), represent the probability of observing *x* spikes emitted by neuron *X*, and *y* spikes by neuron *Y* on the same time window.

All probabilities were estimated by their corresponding frequencies. This approach, also called direct method [35], yields MI values which are biased upward (also called MI naïve). An accurate estimation of the probabilities would require a large number of samples [36]. Practically, we increased the period of the simulations up to 20 minutes, and we noted that after 5 minutes the estimation of the probabilities did not improve much the MI value (less than 4%).

Mutual Information (Equation 1) is symmetric with respect to the exchange of the variables *X* and *Y*. Consequently, it is not suited to recover information on directionality and causality. However, by evaluating MI of time-delayed time series (e.g. by taking *X* as the reference spike train and *Y* as the delayed spike train), we build a MI function of the time-shift, thus providing information both on the synaptic strength and on causality [17], [37]. The peak of each MI function was used to create the CM, so the highest MI values were associated to the strongest connections. Directionality, instead, was determined, as for the CC function, by evaluating the latency of the peak value. Figure 2D shows an example where the MI function presents a peak close to time zero.

#### Joint-Entropy

Cross-Correlation computed on two neurons is essentially proportional to the amount of spikes present in a time window around a reference spike. Thus, cross-correlation based methods ignore any temporal information of the spike patterns. Temporal information can be accounted for by defining an ISI across a pair of neurons. Considering *X* as the reference neuron (the one which actually makes *Y* to spike), then for each spike of *X*, a subsequent spike of *Y* is considered and cross-inter-spike-intervals were defined as time difference (cISI = *t_Y_−t_X_*, cf., Figure 2B).

This section deals with an entropy measure of the cISIs, called Joint-Entropy (JE), defined as:
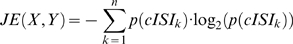
(2)where *p*(cISI*_k_*) is the estimated probability of cISI*_k_*. The cISIs were binned using a bin size equal to 0.1 ms, and cISI_k_ was calculated as k*binsize. The rationale of JE lies in assuming that if *X* and *Y* are strongly connected, then the cISI histogram will show a peak at a specific cISI value, and JE will be close to zero. Conversely, when *X* and *Y* are not connected or weakly connected, the cISI histogram will be nearly flat and consequently JE will be high.

We computed cISI as follows: for each reference spike (*x*), the closest subsequent spike (*y*) is considered; if it falls before a new reference spike, then cISI is computed as their time difference, otherwise it is not accounted. Despite other approaches are also possible (e.g. when two reference spikes are followed by just one spike two cISIs could be computed), the method we used yielded the best results. JE provides asymmetric values, and thus it may be used to infer causality. Differently from the other methods here presented, the strongest connections noticeable in the Synaptic Weight Matrix should be associated to the lowest JE values.

#### Transfer Entropy

Transfer Entropy (TE) is an information theoretic measure which allows to extract causal relationships from time series [18]. It shares some of the desired properties of the Mutual Information (MI), and also it takes into account the history and the dynamics of the peak trains. Differently from MI, TE is not symmetric with respect to the exchange of the variables *X* and *Y*. Additionally, with respect to cross-correlation based methods, TE is sensitive to linear as well as non nonlinear causal interactions [38]. It seems therefore a promising technique to infer connectivity maps.

If we indicate with *x_t_* the number of spikes (of the spike train *X*) falling in the time window *t* (*t* being discrete), then:
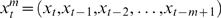
(3)is the spike count vector of the past *m* time windows. Considering a second spike train *Y* and its spike count vector 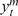, TE can be defined as:
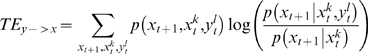
(4)


Mathematically, TE can be interpreted as a measure of the deviation from the generalized Markov property:
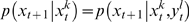
(5)where *p* denotes the transition probabilities conditioned to the past *k* and *l* observations of the spike trains *X* and *Y*, respectively. Low TE values indicate that 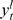 has no influence on the transition probabilities of the state of *X*, so that the assumption of a Markov process holds. On the other hand, high TE values indicate the spike train *Y* influences the response of *X*.

TE can also be written as [30]:
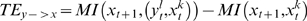
(6)


Equation 6 states that TE measures the gain in information of knowing the future and the past of *x_t_*, once 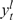 is known.

The probabilities defined by Equation 4 were estimated from the relative frequencies. As for MI computation, we tested the reliability of the estimate by computing TE on longer simulation time windows (from 5 to 20 minutes long). The smallest simulation time window (5 minutes) turned out to be enough to yield an unbiased TE.

Hence, as in other works [18], we restricted our analysis to the case *k* = *l* = 1 (see also the computational considerations reported in “Discussion and conclusions”). The bin and the window size were selected basing on an optimization process. Bin size = 0.3 and window = 1 bin turned out to be optimal in terms of ROC performance (cf., “Bin choice for the parametric methods” into the Results section). Then, the connectivity matrix was built by evaluating the TE for each possible pair of peak trains.

### Validation Procedures

The connectivity methods introduced in the previous section work well when applied on networks made up of few neurons (5–10 neurons) and are almost independent of the synaptic strength and the number of connections. However, in more realistic networks and under increased connectivity conditions, the performance of the connectivity methods rapidly decreases. To validate and quantify the performance of these methods on simulated networks, we used the receiver operating characteristic (ROC) curve [18] and a new method that we called Positive Precision Curve (PPC). All the results presented in this paper were obtained by ignoring the negative weights of the Synaptic Weight Matrix which are associated to inhibitory synapses. Finally, it is worth noting that all the reported error bars represent standard deviation values.

#### Receiver Operating Characteristic–ROC

As stated in the introduction, the Receiver Operating Characteristic (ROC) curves were used to compare the performance of CC, JE, TE and MI. To better appreciate the comparison among these methods, we reduced the ROC curve to a single scalar value (AUC) representing the obtained performance [26], [39]. Since AUC represents the area of a portion of the unit square, its value will be always between 0 and 1. However, since random guessing produces the diagonal line between (0, 0) and (1, 1), which has an area of 0.5, a classifier should have an AUC higher than 0.5 (good classifiers should have AUC values close to 1).

A ROC curve was obtained by comparing the Synaptic Weight Matrix (SWM) and the Thresholded Connectivity Matrix (TCM). For a given threshold, all TCM elements were considered as possible functional connections. If one of the TCM elements corresponds to an existing connection (a non zero value in the SWM), it is considered as a True Positive (TP) and if the connection corresponds to a zero value in the SWM, then it is considered as a False Positive (FP). Furthermore, the TCM elements equal to zero either correspond to an existing connection (a non zero value in the SWM), called False Negative (FN), or they correspond to a null element, called True Negative (TN).

Figures 3B shows the comparison between SWM (left) and CM (right) for the simple neuronal network of Figure 3A.

By changing the threshold (Figure 3D), a variable number of TPs (red circle), FPs (yellow circle), TNs (black circle) and FNs were obtained. Finally, they are reported on a two-dimensional plot by using the following definition of true positive rate (TPR) and the false positive rate (FPR):
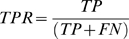
(7)

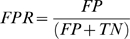
(8)


One of the major limitations of ROC curves is that they do not contain, explicitly, the information regarding the number of TP and FP and that good AUC values (>0.7) can be associated to low ratios of TP vs. FP (TPs<FPs). On the other hand, in order to evaluate the connectivity method performances, also the variations of the TPs and FPs values, as a result of changing the threshold in the TCM, have to be taken into account. As an example, at the beginning of the thresholding procedure of a network characterized by N connections, the FNs are limited by the actual number of connections (FN<N), while the TN value is very high (TN>>N). Thus, by increasing the threshold, the FPR initially will remain close to zero (TN is high) but the TPR will increase more rapidly because of the limited number of FNs, according to Equations 7 and 8. For these reasons, ROC curves alone are not suitable to give a complete picture on the actual performance of the connectivity estimation methods, thus, motivating the introduction of a complementary evaluation method: the PPC.

#### Positive Precision Curve–PPC

In addition to ROC curves, the sensitivity and specificity curves [26] are also used to quantify the performance of classifiers. However, in the context of functional connectivity, the performance of a connectivity method can be stated differently from the context of classifiers. For a given connectivity method, what effectively matters, is its capacity to properly detect a connection, so that mainly TPs (a connection properly detected) and FPs (a connection wrongly detected) have to be taken into account. To this aim we introduced the PPC. We defined the quantity true false ratio (TFR) as:
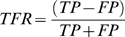
(9)which represents the percentage of TPs detected relative to the FPs. Then, we defined the true-false sum (TFS):
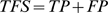
(10)which corresponds to the number of elements being analyzed.

PPC is a 2D graph with TFS on the x-axis and TFR on the y-axis. The PPC was built by considering the TFS-highest values of both the SWM and the CM. An explanatory case is depicted in Figure 3, where panel B shows SWM and CM of a hypothetical simple neuronal network (Figure 3A). The PPC in Figure 3C was built by comparing the sorted SWM and CM values, from the highest to the lowest (white squares, cf., Figure 3D). The sorting process can also be regarded, differently from ROC, as a double threshold process applied to both the SWM and CM. An increase in the TFR value corresponds to an increase of the TP vs. FP ratio. Moreover, since the PPC plot was obtained by an ordered comparison (Figure 3D) of SWM and CM (both thresholded), it is also possible to recognize the capacity of the connectivity methods to detect the right ordering (PPC slope) of the synaptic weights. The PPC slope, in this case, provides information about the performances of the connectivity method as well as the right order in the identification of the connections.

An ideal connectivity method should follow the red curve depicted in Figure 3C, so that TFR is equal to 1 till TFS reaches the maximum number of connections actually present in the SWM. In a real case, the curve would behave more like the blue one (cf., Figure 3C). For instance (Figure 3D), when we consider just one element (TFS = 1), the connection is not detected (TFR = −1); when we include a second element (TFS = 2), two connections of the SWM are properly identified (Figure 3C) and TFR reaches 1.

The PPC peak corresponds to the best trade-off between TPs and FPs detected by the method. Also, the position of the peak is relevant because the identification of a minimum number of connections is necessary to plot a functional connectivity map. To better exemplify, let us consider a network characterized by N = 600 connections with the peak of the PPC equal to 0.2. If the peak is found in correspondence of TFS = 50, it means that the connectivity method identifies 30 TPs and 20 FPs and if the peak position is close to TFS = 500, it implies the identification of 300 TP and 200 FP. Therefore, the value of the PPC peak (TPR) provides information on the ratio between TPs and FPs, while the knowledge of the peak position (TFS) allows identifying the absolute number of TPs and FPs.

Therefore, PPC helps to determine the threshold values to be used for building the connectivity maps. Practically, the chosen threshold value defines the number of selected connections (TFS) and also the performance level (TFR). Since we are interested in selecting a number of links close to the actual number of connections (N) and to maximize the TPs vs. FPs ratio, a good trade-off can be achieved by choosing a TFS between the PPC peak and the estimate of the real number of connections (N).

## Results

In this section, we present the performances of TE, MI, JE and CC evaluated by means of ROC and PPC curves. We applied these methods on two different simulated neuronal networks: namely a network where only excitatory synapses are present (*E* model), and a network which includes also inhibitory connections (*EI* model). Firstly, we showed that the best results, for both the models, are obtained by applying TE. Then, we showed that JE gives rise to better performances with respect to CC when applied to the more realistic *EI* model and, vice versa, that CC gives rise to better results than JE on the simplified *E* one. Finally, we showed that MI gives rise to the worst performances for both the models: a possible interpretation of this behavior is proposed. The methods which provided the best results (TE, JE, and CC) were then applied to the experimental data recorded from *in vitro* cortical neuronal networks coupled to MEAs.

### Evaluation of the connectivity methods performance by means of ROCs and PPCs curves

**Figure 4:**
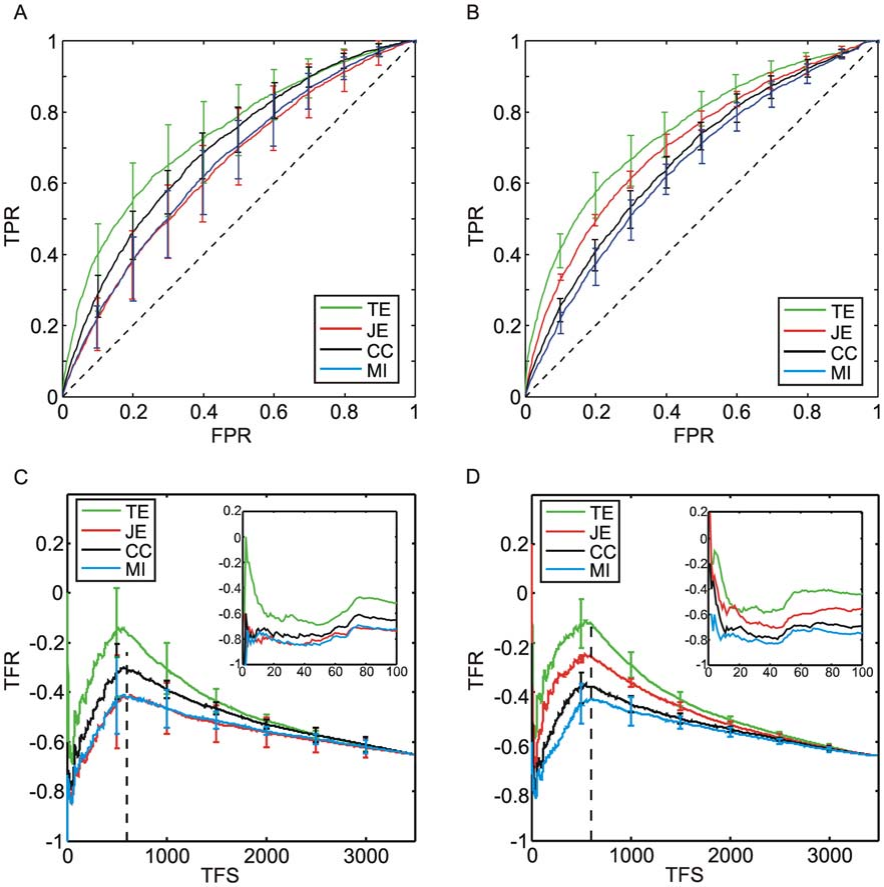
ROC and PPC curves relative to the presented connectivity methods. (A) ROC curves relative to the completely excitatory network models. (B) ROC curves relative to the network models where the connections are both excitatory and inhibitory. Black, blue, red, and green lines indicate CC, MI, JE and TE, respectively. The diagonal (dashed) line (A–B) corresponds to the random detection. (C) PPC evaluated on the *E* network models. (D) PPC evaluated on the *EI* network models. The insets (C–D) show a zoom of the first 100 TFS. The vertical dashed line (C–D) identifies the number of the excitatory elements present in the SWM.

Figures 4A and 4B, respectively, show the ROC curves concerning all the realizations of the excitatory (*E*) and excitatory-inhibitory (*EI*) networks. From the standard error bars (Figures 4A and 4B) we can state that stability is a common feature for all the methods: in fact, we estimated variability between 0.019 and 0.092. The PPCs for *E* and *EI* models are indicated in Figures 4C and 4D, respectively.

ROCs and PPCs concerning *EI* models (Figures 4B and 4D) were evaluated comparing the CM and SWM matrices by considering only the excitatory connections. When also inhibitory connections were considered, ROCs and PPCs showed a systematic reduction of performances. In particular, the decrease was proportional to the performance value estimated on models where inhibitory connections were not considered: TE decreased by 10%, JE by 7%, CC by 2% and MI about 1.5%. Since inhibitory connections cannot be identified by our approach, all the presented analyses were performed just on excitatory connections.

At a first glance, it is possible to note that PPCs (Figures 4C and 4D) report the same macroscopic results already shown by the ROCs (Figures 4A and 4B): TE shows the best performances for both the *E* and *EI* models, while JE displays a good trend only for the *EI* models, and CC only for the *E* ones. Statistical tests (one-way ANOVA, Bonferroni test for means comparison) performed on the 5 considered realizations of *EI* models, indicate that the mean difference of the PPC peaks is significant (except for the couple CC-MI) at the confidence level of 0.05. The same statistical tests indicate a significant AUC mean difference only between TE-CC and TE-MI (confidence level of 0.05). On the other hand, there are no significant differences as far as the *E* models are concerned.

However, from a deeper analysis, it results that, although ROC curves show an apparent positive behavior for all the methods (they are all above the diagonal), PPCs clarify the real and effective percentage of the synaptic connections properly identified. In particular, PPCs fall below zero for all the considered methods (i.e., the number of identified FPs is always greater than the number of TPs). The best performance was obtained by the TE method (Figure 4D) for TFS ≅ 600 and TFR≅−0.1 which corresponds to 270 TPs and 330 FPs.

Moreover, the curves show that the connectivity strengths are not properly identified by the connectivity methods. For instance, if we compare the insets of Figures 4C and 4D with Figure 3C, it appears that the methods fail in detecting the right strengths ordering already for the strongest connections (small TFS values).

In addition, by moving from *E* to *EI* models, the performances of JE improve, whereas they get worse for CC and MI. The mean AUC values, reported in Table 2, support and confirm these considerations. Furthermore, these values indicate TE as the best connectivity method and show a slight improvement of the TE performances on *EI* models.

**Table 2:** AUC (Area Under Curve) values (mean±standard deviation) for the different connectivity methods.

Models	TE	JE	CC	MI
E	0.74±0.09	0.64±0.09	0.70±0.05	0.65±0.07
EI	0.75±0.04	0.71±0.02	0.67±0.04	0.65±0.04

The MI method shows the worst performances (AUC values, Table 2): in particular, on the *EI* models, MI is characterized by bad performances in comparison to other methods. However, on small neuronal networks, characterized by few connections and neurons, MI shows performances comparable to the CC ones (data not shown). An increase in the number of connections and neurons causes the bell-shape of the MI function (Figure 2D) to become flatter while the shape of the CC function (Figure 2C) changes to a lesser extent. The enlargement of the MI bell-shapes causes similar peak values which impairs a proper identification of the strength and of the causality in both *E* and *EI* models.

The high degree of connectivity of actual networks suggests the reasons why MI fails in correctly identifying the connections. Since MI is sensitive to all higher order correlations (both linear and nonlinear), this measure is highly sensitive to a “general influence” caused by the high degree of connectivity. The presence of a “general influence” is also confirmed by the distribution of the Inter Event Intervals (IEIs) evaluated on *EI* models (cf., next section).

On the other hand, although TE method detects also nonlinear correlations (similarly to MI), it displays good performances because it accounts for the past history of each single neuronal activity, thus allowing TE to better distinguish the causal effects between two particular neurons among the “general information flow”.

### Bin choice for the parametric methods

**Figure 5:**
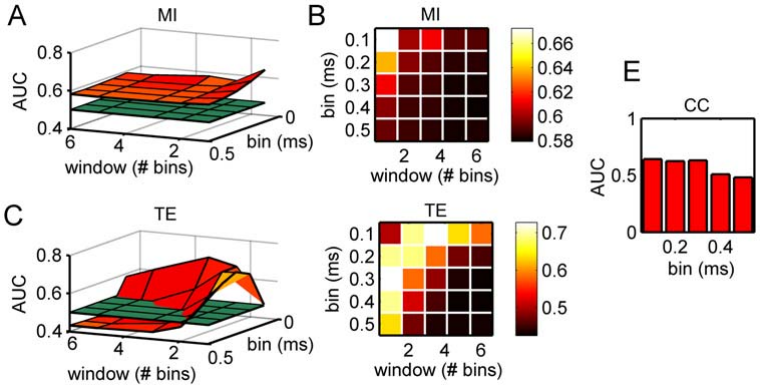
Evaluation of the AUC as a function of the bin size and of the temporal window. (A–B) 3D and false color map representation of the AUC obtained by using the MI method. (C–D) 3D and false color map representation of the AUC obtained by using the TE method. (E) Bar plot of the AUC obtained by using the CC method.

As mentioned in the previous section, CC, MI, and TE methods can be defined as parametric methods: the results obtained by applying such methods depend on the choice of the bin size and temporal window widths. Thus, in order to find the best working conditions, i.e., the set of parameters which maximize the AUC, we tested such methods on the synthetic spike trains generated by a particular realization of the *EI* model. We swept the values of the bin size from 0.1 to 0.5 ms, in a 0.1 ms step, and we considered values of the temporal window made up of 1, 2, 3, 5 and 6 bins. Figure 5 shows the obtained results.

By comparing the performances of the MI (Figure 5A and 5B) with the TE (Figure 5C and 5D), two main results were found. AUC related to MI is always above the critical value of 0.5 (i.e., random choice, represented by means of a green plane). This means that for every bin size and temporal window choice, the MI function yields values which range from 0.583 (bin = 0.5 ms, window = 6 bin) to 0.673 (bin = 0.1 ms, window = 1 bin). By inspection of Figure 5A, we can also appreciate a flat trend except for the tiny bins and temporal windows, where the curve grows and a weak peak is observed. Differently, TE is more sensitive to the choice of the parameters. For a wide range of parameters, the AUC is below the critical value of 0.5 (AUC_min_ = 0.427, bin = 0.4 ms, window = 5 bin): as depicted in Figure 5B, for large bin size and temporal window, the AUC does not show acceptable performances. By decreasing the bin width and the temporal window, the curve overcomes the 0.5-plane (random choice), and for the couple of values 0.1 ms and 3 bins, the TE shows a maximum (AUC_max_ = 0.728), greater than the one obtained for the MI. Therefore, for all the analyses and for both the methods, we used a bin size of 0.1 ms and a temporal window of 3 bins.

The choice of a temporal window of 0.3 ms (3 bins of 0.1 ms) for the TE is compatible with the dynamics found in the neuronal preparations [4]. To support the assumption that such a temporal scale (0.3 ms) is sufficient to investigate the information transmission, we evaluated the Inter Event Interval (IEI). The IEI can be defined as the probability density of time intervals between successive spikes occurring at all the neurons of the MEA/model. Computing the average value of the IEI distribution, we obtained an estimation of the average time between two successive activations of any pair of neurons in the array/network. By evaluating the IEI over 5 realizations of the *EI* model, we found an IEI_AVG_ = 0.35±0.06 ms, which corresponds to the choice of the temporal window used to estimate the TE.

We performed the same analysis on the CC function, which is characterized by only one significant parameter, i.e., the time shift (cf., Cross-Correlation). Thus, we represented in the bar plot of Figure 5E the AUC as a function of this parameter. The result indicates a time shift equal to 0.1 ms for the maximum AUC value (0.642).

### Connectivity Maps from Experimental Data

TE, JE and CC were used to infer and build the functional connectivity maps from experimental data. By applying different thresholds (TCM), the strongest links can be identified and then plotted. In this section, we report the connectivity maps of a cortical network recorded by means of MEA after 26 DIVs.

**Figure 6:**
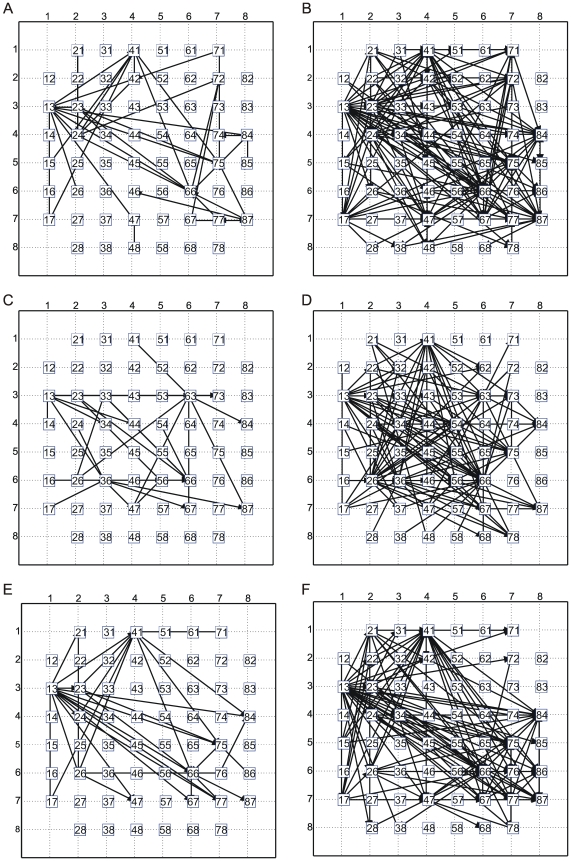
Functional connectivity maps. Connectivity maps obtained on experimental data (MEA) by (A–B) TE, (C–D) JE, (E–F) CC. The threshold values correspond to 40 and 200 links respectively.

Figure 6 show the maps obtained by considering different numbers of connections (i.e., 40 and 200) by using TE (Figure 6A–6B), JE (6C–6D) and CC (6E–6F) methods. It should be underlined that we limited the number of detected connections up to 200 for sake of clarity. Considering the PPCs of the model networks (Figure 4C and 4D), they suggest to select about 600 links to maximize the performance of the connectivity methods. The position of the PPC peak is reminiscent of the connectivity level of the considered models: it is located near the number of connections actually present in the SWM of the model.

Similarly to the network models, we expect to maximize the performance of the connectivity methods by selecting a number of connections near the number of direct connections of a hypothetic SWM of the culture. Marom and Shahaf [9] estimated the average connectivity level of these networks claiming that at their mature phase each neuron is mono-synaptically connected to 10±30% of all the other neurons; these percentages were implemented in our developed models. Therefore, by considering the information contained in the PPCs shown in Figure 4, we could expect to maximize the performance of the connectivity methods by choosing about 600–700 connections also on the experimental data.

By comparing the maps shown in Figure 6, we can notice the presence of some common connections. In particular, the connectivity maps inferred by TE and CC (Figure 6A–6B and 6E–6F) show the most similarity, indicating the most promising methods to be used to estimate connectivity on these experimental preparations.

**Figure 7:**
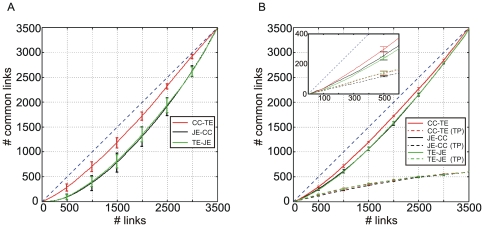
Overlap curves show the number of common links identified by different connectivity methods. (A) Overlap curves from experimental data, (B) overlap curves from the synthetic dataset (mean±standard deviation). The dashed curves show the number of TP values commonly identified by both the methods. The inset highlights the first 600 connections.

In order to better compare the inferred connectivity maps evaluated by means of TE, JE and CC, Figure 7A indicates the number of connections commonly identified on the experimental data considering the methods between pairs. TE and CC (red curve) are characterized by the highest number of common links while CC-TE (black) and JE-TE (green) show similar results. For example, considering 1500 links, TE-CC identified about 1200 common connections, and CC-TE and JE-TE about 800. Moreover, the similarity between TE and CC is also reflected by the correspondence of the optimal threshold value (around 500/700 links).

For comparison, we built also the overlap graph for the simulated dataset coming from the *EI* neuronal network model (Figure 7B).

As expected, TE and CC are characterized by the highest number of connections commonly identified, and the similarity between the results reported in Figure 7A and 7B can be interpreted as a further confirmation of the validity of using these two methods for estimating functional connectivity.

In Figure 7B, we plotted the number of TP values commonly identified by both the methods (dashed lines). In the inset of Figure 7B, a zoom of the first 600 connections is reported. It should be noted that although TE and CC (red curve) identify the highest number of common connections, CC-JE (black curve) and in particular TE-JE (green curve), identified a similar amount of common TPs. Thus, it emerges that TE and CC identify several common FPs. Starting from this observation, we can hypothesize that TE and CC identify also some common indirect causal effects really present in the network which are classified as FPs because they are not accounted by the SWM, where only the direct links are explicitly represented.

### Firing characterization of the neuronal networks

In order to further characterize the firing patterns of the neuronal network models and the experimental data, we performed additional analysis on the ISI distributions.

**Figure 8:**
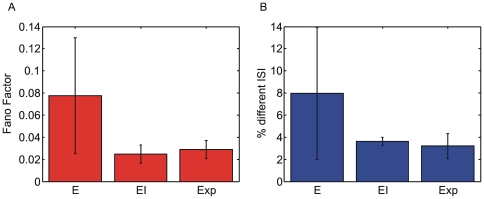
ISI parameters evaluated over simulated and experimental data. (A) Fano Factor evaluated on the probabilities values (y-axis) of the ISI histogram. ISI bin size of 0.2 ms. (B) percentage of non-null ISI bins with respect to the total ISI number.

The ISIs of the *E* model, *EI* model and experimental data were binned by using a bin size of 0.2 ms to build the ISI histograms. To summarize the information contained in these histograms, we evaluated both the spread of the density distribution values (*y* histogram axis) measured in terms of the Fano Factor (FF) [40] and the non-null ISI percentage (the non-null bins relative to the total number of ISIs). The FF is computed on the ISI distribution by evaluating the mean and the variance among the networks ISIs. It is defined as the ratio between the variance 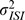 and the mean 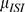 of the ISI distribution. The analyses reported in Figure 8 concern 5 realizations of the simulated models (both *E* and *EI*) and 5 experimental sessions.

The FF was computed on the density distribution values, so that equally distributed ISIs correspond to a FF = 0. Therefore, the definition of the FF on the density distribution values, rather than on the density distribution, implies that small (big) FF values correspond to a flat (peaked) distribution. Figure 8A shows that the ISI distribution of the *EI* model is more spread than the *E* one and closer to the experimental data.

On the other hand, Figure 8B shows the percentage of non-null ISI (with respect to the total number of ISI occurrence) decreases when the network complexity increases (*EI* vs. *E* model). Interestingly, the percentage of non-null ISI in the *EI* models (Figure 8B) well matches the experimental ones. A higher percentage of non-null ISI implies a higher variability in the number of different ISIs and a higher variability in the neuronal firing patterns.

The percentage of non-null ISI and the FF allows quantifying the firing properties of a given neuronal network. A smaller FF and a smaller percentage of non-null ISI means that the firing activity in the *EI* model is more structured than in the *E* one. As it could be intuitively expected, we can deduce that the presence of inhibitory connections plays an important role in structuring the firing dynamics of the models by influencing the interaction among single neurons [41], [42].

Further, the error bars (Figure 8A and 8B) show that completely excitatory models are characterized by a high variability among different *E* realizations, while *EI* models and experimental data are characterized by more similar one. Thus the analyses performed on the ISI distributions (Figure 8A and 8B) allow to conclude that the *EI* model better mimics the dynamics exhibited by the experimental data and, therefore, confirming the plausibility of validating the connectivity methods on the *EI* models.

### Discussion and conclusions

In this work we compared the performances of well established and novel techniques to estimate the functional connectivity in cultured cortical neurons. Although in the literature several papers deal with the identification of functional connections, they usually consider very simplified working conditions (linear time series or networks of few neurons) where the connectivity methods obtain optimal performances for cross-correlations or information theory-based methods [43]. In those cases, considering simplified simulated configurations, visual comparisons between effective connectivity and functional maps were directly performed. Nevertheless, in these conditions, it is also possible to assess whether the observed correlations derive from either direct or indirect connections, or result from a common input.

In our work we extended this approach by testing standard methods (CC, MI, TE) and a novel one (JE) to more realistic and highly-connected simulated networks and by testing the best techniques on actual data obtained from electrophysiological recordings of cultured neurons coupled to MEA devices.

We quantified the performance of four selected connectivity methods by means of Receiver Operating Characteristic (ROC) curves and of a new function named Positive Precision Curve (PPC). Although ROCs curves are widely used, their capability of evaluating the performance of a given method is practically reduced to a single scalar value defined as the Area under the Curve (AUC). This value is useful to have a synthetic outlook of the methods capability to identify network connections but, on the other hand, it does not allow quantifying how many True Positives (TP) and False Positives (FP) there are. On the other hand, PPCs not only allow evaluating the absolute number of the existing/non-existing connections correctly identified by the connectivity methods, but also provide further information regarding how the connectivity methods identify them. In fact, by analyzing the PPC slope, it is possible not only to recover the number of TPs and FPs, but also to investigate if the identified links follow the right synaptic weight ordering. Further, PPC proved to be a reliable technique for selecting the threshold value for maximizing the performances of a specific connectivity method. Similarly to what obtained on the network models, where the position of the PPC peaks (Figure 4C and 4D) are located near the number of the connections effectively present into the models, we expect to maximize the performances by selecting a number of connections close to the estimated synaptic ones actually present on the experimental data (about 600/700 connections).

The first introduced method was CC, one of the most common analytical tool used to study the joint activity of neurons [34], [44] and widely utilized for analyzing synchronized patterns of activity in neuronal cell assemblies. Summarizing the results, we can conclude that CC is a reliable tool with reasonable performances in many contexts. Nevertheless, in our particular application, the degradation of the performances on the *EI* models can be interpreted as a major drawback of the method to work well on data collected by highly connected neuronal cultures. The main problem of the CC method is that linear dependencies are unlikely to govern such neuronal connectivity.

Mutual Information (MI) [45] is a mathematically rigorous approach for the detection of the interdependences between time series, and it is widely used in many fields (e.g., telecommunication, machine learning); differently from CC [46], MI depends on all higher order moments of the probability distribution. Therefore, MI measures are not restricted to the second order (as the CC ones), but they are sensitive to all higher order correlations, both linear and nonlinear. Since MI is a symmetrical measure, it cannot detect directional flow of information and causal relationships. In order to overcome this limitation, we built, for each pair of neurons, a MI function by delaying the peak trains. Although the introduction of a time delay allows good connectivity maps to be obtained on small networks, on large and highly connected networks MI performances decreases. In fact, MI showed the worst performances on *E* and *EI* models and, as a consequence, on the experimental networks. The general influence among neurons, due to the high density of the cultures, makes MI unable to accurately detect causal relationships on complex models and on experimental data.

Two entropy measures were also considered. Transfer Entropy (TE) [38], [47] is a recent tool for investigating neuronal assemblies and for quantifying the fraction of neuron information found in the past history of another one [43]. Since TE estimates the part of activity of a neuron that is not dependent on its own past but on the past activity of another neuron, the obtained results are likely to be more precise with respect to the ones provided by other methods. Moreover, TE takes into account linear and nonlinear interactions and thus may represent a very general way to define the causality strength between two spikes trains. Considering the results here presented, TE showed the best results both on *E* (Figure 4A and 4C) and on the *EI* models (Figure 4B and 4D). TE is then a good estimation method which can be conveniently applied also on real datasets.

Joint-Entropy (JE) is a novel measure of entropy which was applied for the first time in the field of neuroscience. Analyzing purely excitatory networks, JE shows the worst performances (similarly to MI), while it provides interesting results with inhibitory-excitatory networks. Considering the results presented in Figure 8, pointing out the similarity between the experimental data and the *EI* model, and considering that JE measures are computed based on cISI distributions [20], [21], it turns out that JE is sensitive to the activity patterns showed by the neuronal networks and is capable to distinguish the influence of a specific neurons on the activity of another one. For these reasons, despite its simplicity, the JE measure can be considered as a good alternative method to TE to be applied to real data.

In order to better evaluate the efficiency of these connectivity methods, it is also interesting to know the performance in terms of required computational time. All the tests were carried out on an INTEL Quad Core, clock 2.83 GHz, RAM 4 GB, by using the same dataset and parameter values reported in the paper. JE proved to be the fastest method. Considering a 5 minutes simulation, JE method took around 15 minutes to analyze the data. CC, TE, and MI took respectively 30 minutes, 16 hours and 2 days. Next, we extended the same analysis to longer simulations, lasting from 5 to 30 minutes. The time needed to accomplish the same analysis increased linearly for all the proposed methods with increasing slopes of 4, 12 and 200 for JE, CC and TE respectively. MI was not included in this analysis because of its bad performances and prohibitive time needed to process the data. The qualitative evaluation of the goodness/efficiency suggests JE as an optimal trade-off method especially for recordings including a very large number of neurons.

The overlap curves (Figure 7A), which underline the number of common links identified by the different methods, are the only possible feedback regarding the performance of the connectivity methods when applied to experimental data. By observing these curves, we can note the good agreement between TE and CC, as in the case of model networks (Figure 7B). Interestingly enough, this agreement is not restricted to the TP values: TE and CC identify also a high number of common FPs. From this, we can speculate that TE and CC recognize some strong indirect connections actually present on the neuronal network but not classified as TPs because only direct links are represented into the Synaptic Weight Matrix.

The approach we adopted, by considering the connection strength proportional to the value of the connectivity method, does not allowed us to identify inhibitory connections. A previous report [21] showed that inhibitory connections could be detected by looking at the time shifts of the cISI histogram built on the cross activities of two neurons. However in the *EI* network models we observed that the cISI histograms built on neurons contacted by zero to up to 5 inhibitory neurons did not showed any significant time shift. We therefore believe that the collective highly synchronous and bursting activity across the whole network prevents to recover inhibitory connections by simply looking to pair wise activities.
